# Clinical characteristics and the role of IL-6 in acute-on-chronic liver failure patients with or without COVID-19: a multicenter paired cohort study

**DOI:** 10.3389/fcimb.2024.1471974

**Published:** 2025-01-10

**Authors:** Ruoyu Yao, Guofen Xu, Xiujuan Fu, Wenrui Zhang, Han Wang, Yu Chen, Jia Yao

**Affiliations:** ^1^ Department of Gastroenterology, Third Hospital of Shanxi Medical University, Shanxi Bethune Hospital, Shanxi Academy of Medical Sciences, Tongji Shanxi Hospital, Taiyuan, China; ^2^ Fourth Department of Liver Disease (Difficult and Complicated Liver Diseases and Artificial Liver Center), Beijing You’an Hospital Affiliated to Capital Medical University, Beijing, China

**Keywords:** acute-on-chronic liver failure, COVID-19, SARS-CoV-2, mortality, prognosis, IL-6

## Abstract

**Background and Aims:**

The impact of coronavirus disease 2019 (COVID-19) on patients with acute-on-chronic liver failure (ACLF) remains unclear. To investigate the clinical characteristics of patients with ACLF complicated with COVID-19 in order to provide evidence for the precise treatment of this patient population.

**Methods:**

A total of 34 ACLF patients with COVID-19 admitted to these three hospitals from December 2022 to August 2023 were included as the ACLF+COVID-19 group. Additionally, 34 age-, gender-, etiology-, and Model for End-Stage Liver Disease-Sodium (MELD-Na) score-matched ACLF patients were screened from 286 ACLF patients as the ACLF group. From 382 COVID-19 patients, 34 were selected as the COVID-19 group, matching the ACLF+COVID-19 group in age, gender, and illness severity. Clinical features of these three groups were compared, with the primary measure being the 28-day mortality rate in the ACLF patients and the secondary measures including clinical symptoms, laboratory tests, comorbidities, and complications in three groups.

**Results:**

Compared with the ACLF group, the ACLF+COVID-19 group had significantly higher incidence rates of fever, cough, sputum production, fatigue, and hypoxemia (all p<0.01). Patients in the ACLF+COVID-19 group were more likely to have hepatic encephalopathy (p=0.015), lower platelet count (p=0.016) and elevated IL-6 level (p=0.026), and higher MELD-Na score (p=0.041) one week after admission, but without a significant increase in 28-day mortality rate (p=0.16).

**Conclusions:**

ACLF patients with COVID-19 have increased risk for thrombocytopenia, more obvious inflammatory response, and rapid disease progression 1 week after admission, but the 28-day mortality rate is similar to that of ACLF patients without COVID-19.

## 1 Introduction

Acute-on-chronic liver failure (ACLF) is a clinical syndrome characterized by acute liver injury and organ failure in patients with chronic liver disease (CLD) or cirrhosis, with a high risk of short-term mortality (Sarin et al., 2019). Amidst the coronavirus disease 2019 (COVID-19) pandemic, which has emerged as the most significant public health challenge in the 21st century, a growing body of research has documented cases of liver dysfunction in COVID-19 patients (Jothimani et al., 2020 ^;^ Sun et al., 2020). Therefore, the persisting severe acute respiratory syndrome coronavirus 2 (SARS-CoV-2) infection poses a substantial challenge for the management of ACLF patients.

COVID-19 is characterized by respiratory symptoms such as fever, cough, and sputum production (Singh and Khan, 2020), in addition to varying degrees of liver injuries (Cichoz-Lach and Michalak, 2021). A large multicenter study on liver injury patterns of COVID-19 in Asia found that SARS-CoV-2 infection could lead to severe liver injury in patients with chronic liver disease, decompensating one fifth of cirrhosis and worsening the clinical status of these patients (Sarin et al., 2020). In addition, a North American multicenter paired study revealed a poor overall prognosis among patients with cirrhosis and COVID-19 (Bajaj et al., 2021). Studies have shown that SARS-CoV-2 binds to angiotensin-converting enzyme 2 (ACE2) on human cells and triggers immune responses and inflammation, leading to injuries to multiple organs and tissues including the lungs, liver, and muscles (Ashraf et al., 2021 ^;^ Gusev et al., 2022). SARS-CoV-2 has also been reported to induce dysregulated inflammatory responses and trigger cytokine storms (Fara et al., 2020). The progression of ACLF is closely associated with immune dysregulation, and the activation of systemic immune responses and inflammation is widely acknowledged as the central pathogenic mechanism of ACLF (Wu et al., 2010 ^;^ Fyfe et al., 2018). Cytokines play a key role in the progression of ACLF, and the secretion of cytokines, including interleukin-6 (IL-6), triggers extensive hepatocyte death and exacerbates the decline in liver function (Casulleras et al., 2020). However, the impact of COVID-19 on the prognosis of ACLF patients remain unclear, and few studies have compared the outcomes of ACLF with or without COVID-19. As COVID-19 remains an established and ongoing health issue, ACLF patients will continue to be at risk of contracting COVID-19. Further clinical observation and research will help us understand the impact of COVID-19 on ACLF patients and optimize the management of this patient population.

The present multicenter paired study aimed to compare the clinical symptoms, comorbidities, complications, laboratory data, and 28-day mortality rate between ACLF patients with and without COVID-19 and to elucidate the impact of COVID-19 on ACLF patients.

## 2 Methods

### 2.1 Study design and subjects

All patients hospitalized due to ACLF and all SARS-CoV-2-positive patients in Shanxi Bethune Hospital, Beijing You’an Hospital, and Jincheng General Hospital from December 2022 to August 2023 were retrospectively reviewed in this multicenter paired cohort study.

The inclusion criteria were: (i) with a diagnosis of ACLF according to the Asian Pacific Association for the Study of the Liver (APASL) expert consensus on ACLF: acute hepatic insult manifesting as jaundice (TBIL≥85 µmol/L) and coagulopathy (INR ≥ 1.5 or PTA< 40%), complicated within 4 weeks by ascites and/or hepatic encephalopathy (HE) in a patient with previously diagnosed or undiagnosed chronic liver disease or cirrhosis (Sarin et al., 2019); (ii) with a diagnosis of COVID-19 according to the *Diagnosis and Treatment Protocol for COVID-19 (Trial Version 10)*, which requests a positive COVID-19 nucleic acid test or COVID-19 antigen test; and (iii) aged 18 - 70 years. The exclusion criteria were: (i) with other lung infections (e.g., influenza A/B or pulmonary bacterial infection); (ii) with liver cancer and/or other malignant tumors; and (iii) with other disorders of the immune system.

**Figure 1:**
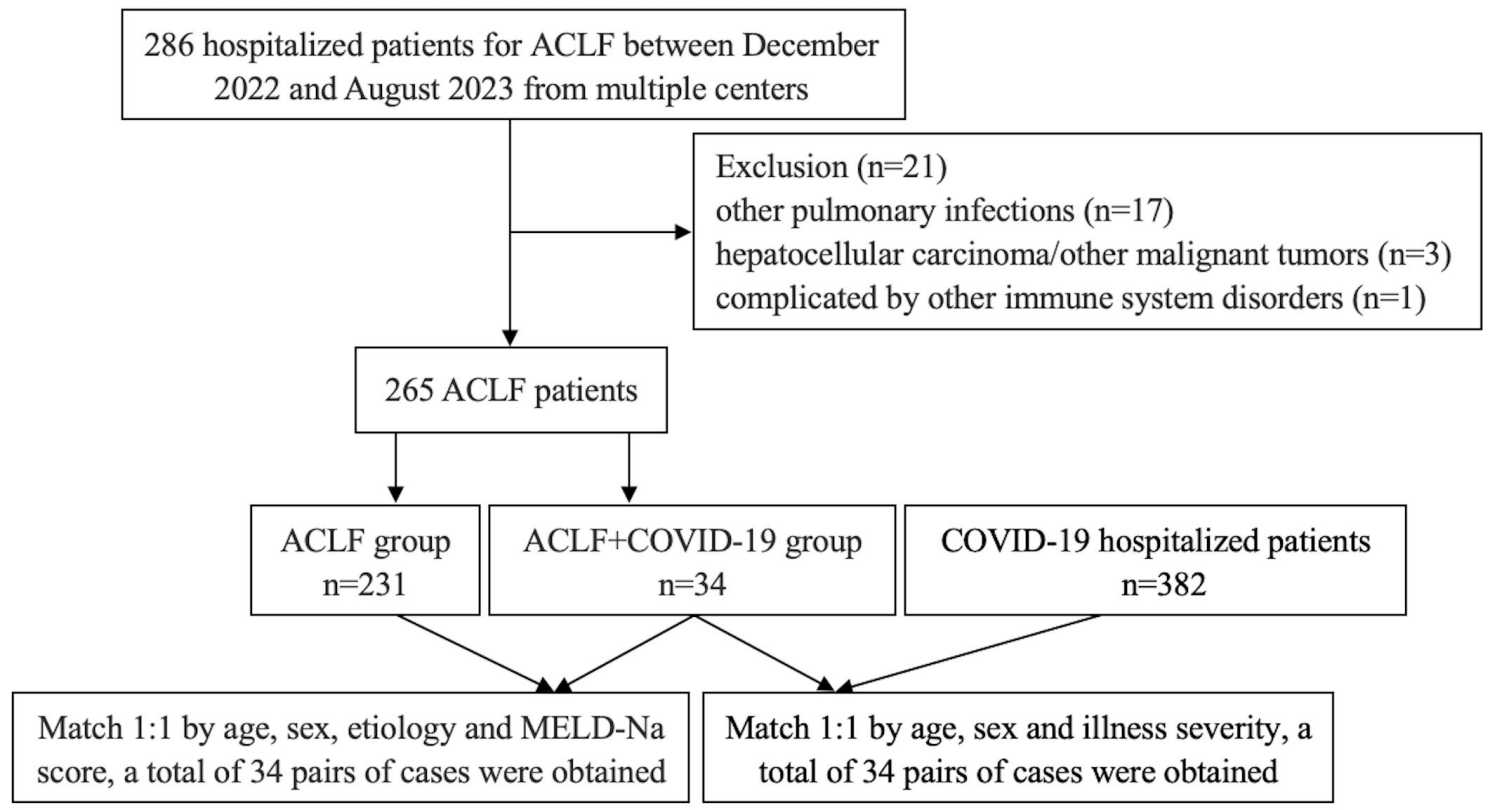
Flowchart of patient screening and pair-matching.

All the included ACLF patients were divided into ACLF+COVID-19 group and ACLF group according to the presence or absence of COVID-19. Patients in these two groups were matched 1:1 according to age, gender, etiology, and Model for End-stage Liver Disease-Sodium (MELD-Na) score at the time of admission. The ACLF+COVID-19 group and the COVID-19 group were matched 1:1 in terms of age at admission, gender, and illness severity (**Figure 1**). The study was conducted in accordance with the Declaration of Helsinki, and the research protocol was approved by Shanxi Bethune Hospital (NO: YXLL-2023-233).

### 2.2 Collection of clinical data

The basic demographic data and clinical information of each patient during hospitalization, including gender, age, etiology, symptoms, comorbidities, complications, and 28-day mortality rate, were collected from the electronic medical records. The laboratory data including blood cell counts, liver and kidney functions, coagulation-related indicators, and cytokines were also collected. The MELD-Na score, APASL ACLF Research Consortium (AARC) score, and Sequential Organ Failure Assessment (SOFA) score at admission and the MELD-Na score after 1 week were calculated. All the collected data were reviewed by the research team and double-checked by experienced physicians.

All ACLF patients received hepatoprotective, enzyme-lowering, and anti-jaundice drug therapy, along with albumin supplementation. General supportive care and nonbiological artificial liver (NBAL) were applied. Molnupiravir was administered in the ACLF+COVID-19 group. Patients in the COVID-19 alone group were given antiviral therapy and general supportive care.

### 2.3 Sample size calculation

With an attempt to investigate the clinical characteristics of ACLF patients with COVID-19, we divided the enrolled ACLF patients into ACLF+COVID-19 group and ACLF group. Preliminary experiments showed IL-6 levels of 155.33 ± 98.1 in the ACLF+COVID-19 group and 51.97 ± 56.48 in the ACLF group. Accordingly, a sample size of 12 patients per group was calculated using the PASS 2021 software (NCSS, LLC, USA) with a significance level (α) of 0.05, a power of 0.8, and an equal allocation ratio of 1:1.

### 2.4 Statistical analysis

Statistical analysis was carried out using the SPSS software package (version 26.0; IBM). The normally distributed measurement data are presented as mean ± standard deviation and compared using independent samples *t* test. The non-normally distributed measurement data are presented as medians (interquartile ranges) and compared using a nonparametric test (Kruskal-Wallis test). Count data are described as frequency or percentage and compared using a Chi-square test or Fisher’s exact test. The survival curves were plotted by the Kaplan-Meier survival analysis method. The Spearman correlation test was used to analyze the potential correlations of IL-6 with MELD-Na score, AARC score, and SOFA score. A *p* value <0.05 was considered statistically significant.

## 3 Results

### 3.1 Basic features of the subjects

The age, gender, etiology, and MELD-Na score were matched between the two ACLF groups. While the incidence of cardiovascular disease (CVD) was similar, the ACLF+COVID-19 group had significantly higher incidence rates of type 2 diabetes mellitus (T2DM) (8 [23.5%] *vs* 2 [5.89%]; *p*=0.04) and hepatic encephalopathy (14 [41.18%] *vs* 5 [14.71%]; *p*=0.015). The total bilirubin (TBIL), direct bilirubin (DBIL), and prothrombin time (PT) were higher than the normal ranges in ACLF+COVID-19 patients, whereas the albumin (ALB) level was below the minimum thresholds. In addition, the ACLF+COVID-19 group had a significantly lower platelet (PLT) count than the ACLF group (55.5 [24-99] *vs* 90 [77-167]; *p*=0.016). Compared with the COVID-19 group, the ACLF+COVID-19 group had significantly higher levels of MELD-Na score (27 ± 13 vs 8 ± 3; p<0.001), aspartate aminotransferase (AST) (113.2 [31.25-236] vs 26.7 [20.8-34.6]; p=0.001), TBIL (263.85 ± 188.11 vs 15.03 ± 5.5; p<0.001), DBIL (173.9 [45.83-371.6] vs 3.2[2.4-4.8]; p<0.001), PT (25.22 ± 5.34 vs 11.8[10.9-12.5]; p<0.001), international normalized ratio (INR) (2.36 [1.87-2.62] vs 1.09 [1.01-1,13]; p<0.001), D-dimer (DD) (1060.5 [309.25-3506.5] vs 169.13 ± 88.73; p=0.001), neutrophils (7.54 ± 5.92 vs 4.27 ± 2.25; p=0.029), and lower rate/levels of CVD (2 [5.89%] vs 13 [38.24%]; p=0.001), ALB (30.07 ± 6.01 vs 38.45 ± 5.03; p<0.001), hemoglobin (Hb) (98.47 ± 32.99 vs 140.27 ± 14.69; p<0.001), and PLT (55.5 [24-99] vs 180 [124-225]; p<0.001) (**Table 1**).

**Table 1:** Basic features of patients in the three groups at admission.

	ACLF group (n=34)	ACLF+COVID-19 group (n=34)	COVID-19 group (n=34)	^a^P-value	^b^P-value
Age, years	52 ± 9.67	52.82 ± 9.89	52.71 ± 10.01	0.73	0.769
Male, No. (%)	23 (67.65%)	23 (67.65%)	23 (67.65%)	1	1
Etiology					
HBV/Alcohol/Drug/AIH/Other, No.	11/16/2/3/2	11/16/1/3/3	–	1	–
Comorbidity					
T2DM, No. (%)	2 (5.88%)	8 (23.53%)	14 (41.18%)	0.04	0.12
Hypertension, No. (%)	8 (23.53%)	6 (17.65%)	12 (35.29%)	0.549	0.099
CVD, No. (%)	1 (2.94%)	2 (5.88%)	13 (38.24%)	1	0.001
Complication					
Ascites, No. (%)	29 (85.29%)	30 (88.24%)	0 (0%)	1	<0.001
HE, No. (%)	5 (14.71%)	14 (41.18%)	0 (0%)	0.015	<0.001
Blood Test Parameter					
ALT, IU/L	137.1 (42.2-377.7)	35.3 (21.93-348.5)	33.69 ± 24.67	0.191	0.165
AST, IU/L	328 (82.3-488.4)	113.2 (31.25-236)	26.7 (20.8-34.6)	0.237	0.001
TBIL, umol/L	390.84 ± 175.58	263.85 ± 188.11	15.03 ± 5.5	0.066	<0.001
DBIL, umol/L	288 (134.7-388.6)	173.9 (45.83-371.6)	3.2 (2.4-4.8)	0.101	<0.001
ALB, g/L	27.92 ± 3.49	30.07 ± 6.01	38.45 ± 5.03	0.242	<0.001
BUN, umol/L	4.2 (3.03-9.53)	8.65 (3.63-15.6)	5.63 ± 1.65	0.291	0.144
Cr, mmol/L	90 (75.4-125.1)	84.75 (70.5-141.3)	91.23 ± 25.84	0.917	0.548
K^+^, mmol/L	3.72 ± 0.8	3.83 ± 0.73	3.77 ± 0.26	0.682	0.761
Na^+^, mmol/L	133.79 ± 6.07	134.69 ± 5.26	136.34 ± 3.62	0.67	0.325
PT, s	22.39 ± 6.81	25.22 ± 5.34	11.8 (10.9-12.5)	0.215	<0.001
INR	1.91 (1.61-2.26)	2.36 (1.87-2.62)	1.09 (1.01-1.13)	0.174	<0.001
DD, ng/mL	1260 (953-3268)	1060.5 (309.25-3506.5)	169.13 ± 88.73	0.359	0.001
WBC, ×10^9^/L	7.12 ± 2.87	9.22 ± 6.04	6.21 ± 2.83	0.234	0.071
Hb, g/L	117.47 ± 30.09	98.47 ± 32.99	140.27 ± 14.69	0.111	<0.001
PLT, ×10^9^/L	90 (77-167)	55.5 (24-99)	180 (124-225)	0.016	<0.001
Neutrophil, ×10^9^/L	5.05 ± 2.54	7.54 ± 5.92	4.27 ± 2.25	0.145	0.029
Lymphocyte, ×10^9^/L	1.13 ± 0.59	0.86 ± 0.67	1.23 ± 0.82	0.254	0.11
Clinical Score					
MELD-Na score	26 ± 8	27 ± 13	8 ± 3	0.932	<0.001
AARC score	10 ± 2	10 ± 2	–	1	–
AARC score grade I	4 (11.76%)	3 (8.82%)	–	1	–
AARC score grade II	20 (58.82%)	20 (58.82%)	–	1	–
AARC score grade III	10 (29.41%)	11 (32.35%)	–	0.793	–
SOFA score	7 (6-9)	8 (7-9)	–	0.215	–

^a^P-value: Comparison between ACLF+COVID-19 group and ACLF group. ^b^P-value: Comparison between ACLF+COVID-19 group and COVID-19 group. HBV, hepatitis B virus; AIH, autoimmune hepatitis; T2DM, type 2 diabetes mellitus; CVD, cardiovascular disease; HE, hepatic encephalopathy; ALT, alanine aminotransferase; AST, aspartate aminotransferase; TBIL, total bilirubin; DBIL, direct bilirubin; ALB, albumin; BUN, blood urea nitrogen; Cr, creatinine; PT, prothrombin time; INR, international normalized ratio; DD, D-dimer; WBC, white blood cell; Hb, hemoglobin; PLT, platelets.

### 3.2 Clinical manifestations of patients with ACLF and COVID-19

The incidence rates of fever (23 [67.6%] *vs* 12 [35.29%]; *p*=0.008), cough (18 [52.94%] *vs* 6 [17.65%]; *p*=0.002), sputum production (15 [44.12%] *vs* 4 [11.76%]; *p*=0.003), fatigue (20 [58.82%] *vs* 8[23.5%]; *p*=0.003), and hypoxemia (12 [35.29%] *vs* 3 [8.82%]; *p*=0.008) in the ACLF+COVID-19 group were significantly higher compared with those in the ACLF group. Patients with ACLF+COVID-19 group had lower rates of cough (18 [52.94%] vs 28 [82.35%]; p=0.01), sputum production (15 [44.12%] vs 27 [79.41%]; p=0.003), dyspnea (8 [23.53%] vs 17 [50%]; p=0.024), and muscle aches (1 [2.94%] vs 10 [29.41%]; p=0.003) compared with those in the COVID-19 group (**Table 2**).

**Table 2:** Clinical manifestations of patients in the three groups.

	No. (%)				
	ACLF group (n=34)	ACLF+COVID-19 group (n=34)	COVID-19 group (n=34)	^a^P-value	^b^P-value
Fever	12 (35.29%)	23 (67.65%)	24 (70.59%)	0.008	0.793
Cough	6 (17.65%)	18 (52.94%)	28 (82.35%)	0.002	0.01
Sputum production	4 (11.76%)	15 (44.12%)	27 (79.41%)	0.003	0.003
Fatigue	8 (23.53%)	20 (58.82%)	20 (58.82%)	0.003	1
Dyspnea	5 (14.71%)	8 (23.53%)	17 (50%)	0.355	0.024
Chest discomfort	2 (5.88%)	4 (11.76%)	9 (26.47%)	0.673	0.123
Nausea/vomiting	7 (20.59%)	6 (17.65%)	5 (14.71%)	0.758	0.742
Abdominal pain	5 (14.71%)	8 (23.53%)	2 (5.88%)	0.355	0.04
Diarrhea	6 (17.65%)	10 (29.41%)	2 (5.88%)	0.253	0.011
Bloating	21 (61.76%)	25 (73.53%)	0 (0%)	0.3	<0.001
Muscle aches	0 (0%)	1 (2.94%)	10 (29.41%)	1	0.003
Runny nose	1 (2.94%)	3 (8.82%)	3 (8.82%)	0.614	1
Sore throat	0 (0%)	4 (11.76%)	8 (23.53%)	0.114	0.203
Dysgeusia	0 (0%)	1 (2.94%)	1 (2.94%)	1	1
Anosmia	0 (0%)	2 (5.88%)	1 (2.94%)	0.493	1
Hypoxemia	3 (8.82%)	12 (35.29%)	13 (38.24%)	0.008	0.801

^a^P-value: Comparison between ACLF+COVID-19 group and ACLF group. ^b^P-value: Comparison between ACLF+COVID-19 group and COVID-19 group.

### 3.3 Inflammatory cytokines

IL-6, IL-8, and IL-10 levels were higher than the normal ranges in both ACLF groups. However, the IL-6 level in the ACLF+COVID-19 group was significantly higher than that in the ACLF group (161.43 [82.75 - 479.9] vs 43.47 [14.35-100.21]; p=0.026). In addition, the ACLF+COVID-19 group had higher IL-6 levels (161.43 [82.75 - 479.9)] vs 7.96 [29.3-18.54]; p=0.004), IL-8 levels (302.77 ± 264.24 vs 7.53 ± 9.24; p=0.041), and IL-17 levels (28.89 ± 24.43 vs 0 [0-2.76]; p=0.022) than the COVID-19 group (**Table 3**).

**Table 3:** Cytokine levels in the three groups.

	ACLF group (n=34)	ACLF+COVID-19 group (n=34)	COVID-19 group (n=34)	^a^P-value	^b^P-value
IL-1β, pg/mL	3.21 ± 0.71	2.46 ± 1.09	2.03 ± 1.61	0.186	0.595
IL-2, pg/mL	2.25 (1.36-3.12)	2.75 (1.56-3)	1.42 ± 0.52	1	0.059
IL-4, pg/mL	3.07 ± 0.66	2.26 ± 1.06	1.08 ± 0.89	0.144	0.063
IL-5, pg/mL	2.21 ± 0.56	1.61 ± 0.44	1.26 (1.08-1.78)	0.66	0.42
IL-6, pg/mL	43.47 (14.35-100.21)	161.43 (82.75-479.9)	7.96 (29.3-18.54)	0.026	0.004
IL-8, pg/mL	388.4 ± 342.02	302.77 ± 264.24	7.53 ± 9.24	0.638	0.041
IL-10, pg/mL	10.47 ± 6.52	14.05 ± 2.85	5.38 (3.36-52.95)	0.246	0.337
IL-12p70, pg/mL	4.35 ± 1.27	2.86 ± 1.23	0.8 (0-4.45)	0.66	0.149
IL-17, pg/mL	20.89 ± 12.16	28.89 ± 24.43	0 (0-2.76)	0.489	0.022
INF-α, pg/mL	2.5 ± 0.88	2.43 ± 1.55	4.79 ± 4.38	0.916	0.257
INF-γ, pg/mL	2.66 (2.55-4.16)	3.15 (1.93-3.82)	2.47 (1-4.77)	0.973	0.422
TNF-a, pg/mL	3.19 (2.17-4.16)	3.08 (1.46-15.9)	1.25 ± 0.8	0.937	0.065

^a^P-value: Comparison between ACLF+COVID-19 group and ACLF group. ^b^P-value: Comparison between ACLF+COVID-19 group and COVID-19 group.

### 3.4 Correlations of IL-6 level with clinical scores

**Figure 2:**
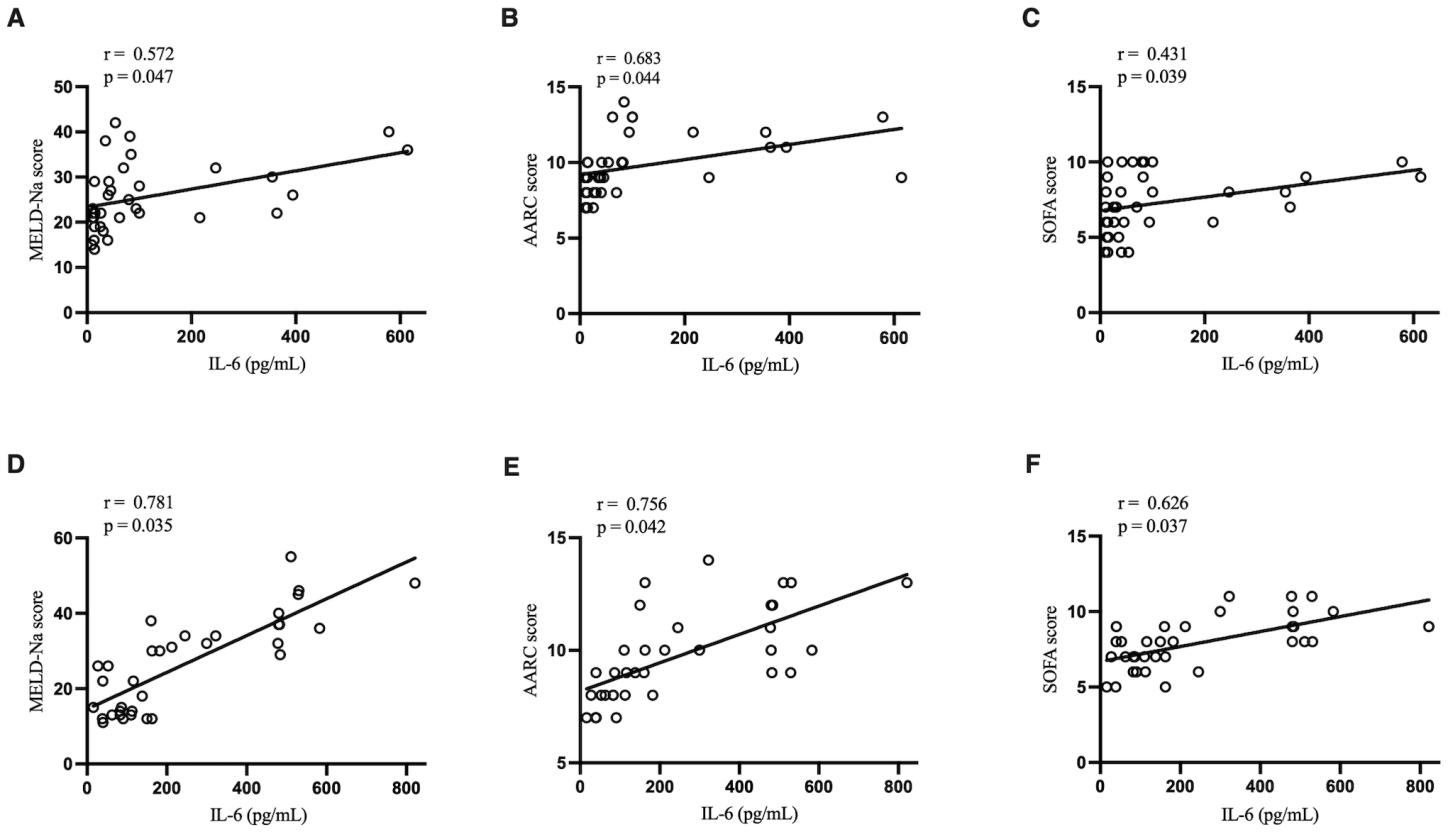
Correlation of IL-6 with MELD-Na score, AARC score, and SOFA score. **(A)** Correlation of IL-6 with MELD-Na score in the ACLF group. **(B)** Correlation of IL-6 with AARC score in the ACLF group. **(C)** Correlation of IL-6 with SOFA score in the ACLF group. **(D)** Correlation of IL-6 with MELD-Na score in the ACLF+COVID-19 group. **(E)** Correlation of IL-6 with AARC score in the ACLF+COVID-19 group. **(F)** Correlation of IL-6 with SOFA score in the ACLF+COVID-19 group.

In the two ACLF groups, IL-6 level had a significant positive correlation with MELD-Na score, AARC score, and SOFA score. Notably, such correlations were stronger in the ACLF+COVID-19 group (**Figure 2**).

### 3.5 Patient features in two ACLF groups after stratification with AARC score

Stratified by AARC score, the IL-6 levels were significantly elevated in the ACLF+COVID-19 patients with an AARC score grade I (80.12 [81.29-82.45] vs 11.15 [13.22-15.28]; *p*=0.018) and II (230.44 [81.87-495.25] vs 36.84 [12.85-81.01]; *p*=0.001) compared with the ACLF group. The incidence of hepatic encephalopathy was significantly higher in patients with ACLF+COVID-19 with AARC score III (9 [81.82%] vs 3 [30%]; *p* = 0.03). Additionally, the ACLF+COVID-19 patients with an AARC score grade II (70 [33.25-108.75] vs 133.5 [83-167]; *p*=0.003) and III (46.18 ± 26.03 vs 74.6 ± 31.83; *p*=0.037) had significantly lower platelet counts (**Table 4**).

**Table 4:** Comparisons of the clinical features between ACLF and ACLF+COVID-19 groups after stratification according to AARC scores.

	AARC score grade I			AARC score grade II			AARC score grade III		
	ACLF group (n=4)	ACLF+COVID-19 group (n=3)	P-value	ACLF group (n=20)	ACLF+COVID-19 group (n=20)	P-value	ACLF group (n=10)	ACLF+COVID-19 group (n=11)	P-value
ALT, IU/L	50.13 ± 42.74	115.08 ± 79.9	0.229	86.45 (33.9-447.08)	51.25 (19.83-441.65)	0.598	281.65 (45.02-453.55)	37.4 (22.1-338)	0.181
AST, IU/L	137.87 ± 153.62	197.83 ± 163.11	0.643	164.65 (44.98-351.68)	113.2 (30.05-208.23)	0.543	443.5 (83.83-543.4)	180.5 (71-681)	0.526
TBIL, umol/L	340.73 ± 223.4	156.91 ± 44.56	0.228	380.75 ± 142.36	281.25 ± 174.69	0.056	431.06 ± 224.68	261.38 ± 233.13	0.106
ALB, g/L	36.90 ± 8.26	31.33 ± 2.54	0.363	27.79 ± 3.73	28.03 ± 5.21	0.871	27.83 ± 3.54	30.86 ± 5.76	0.168
Cr, mmol/L	52.97 ± 17.29	71.55 ± 18.6	0.236	99.65 (82.08-125.7)	86.05 (58.25-131.25)	0.14	89.5 (72.98-12)	90.7 (73-148)	0.622
INR	1.84 ± 0.49	1.77 ± 0.23	0.806	1.96 (1.74-2.9)	2.39 (1.96-2.66)	0.499	1.75 (1.52-2.26)	1.94 (1.88-2.4)	0.091
PLT, ×10^9^/L	230.5 ± 126.68	118.67 ± 104.58	0.271	133.5 (83-167)	70 (33.25-108.75)	0.003	74.6 ± 31.83	46.18 ± 26.03	0.037
IL-6, pg/mL	11.15 (13.22-15.28)	80.12 (81.29-82.45)	0.018	36.84 (12.85-81.01)	230.44 (81.87-495.25)	0.001	134.93 ± 95.78	211.01 ± 158.71	0.386
HE, No. (%)	1 (25%)	2 (66.67%)	0.486	1 (5%)	3 (15%)	0.605	3 (30%)	9 (81.82%)	0.03
Ascites, No. (%)	3 (75%)	2 (66.67%)	1	19 (95%)	18 (90%)	1	7 (70%)	10 (90.91%)	0.311
28-day mortality rate, No. (%)	0 (0%)	0 (0%)	–	0 (0%)	1 (5%)	1	1 (10%)	3 (27.27%)	0.586

ALT, alanine aminotransferase; AST, aspartate aminotransferase; TBIL, total bilirubin; ALB, albumin; Cr, creatinine; INR, international normalized ratio; PLT, platelets; HE, hepatic encephalopathy.

### 3.6 28-day mortality rates in the ACLF+COVID-19 group

**Figure 3:**
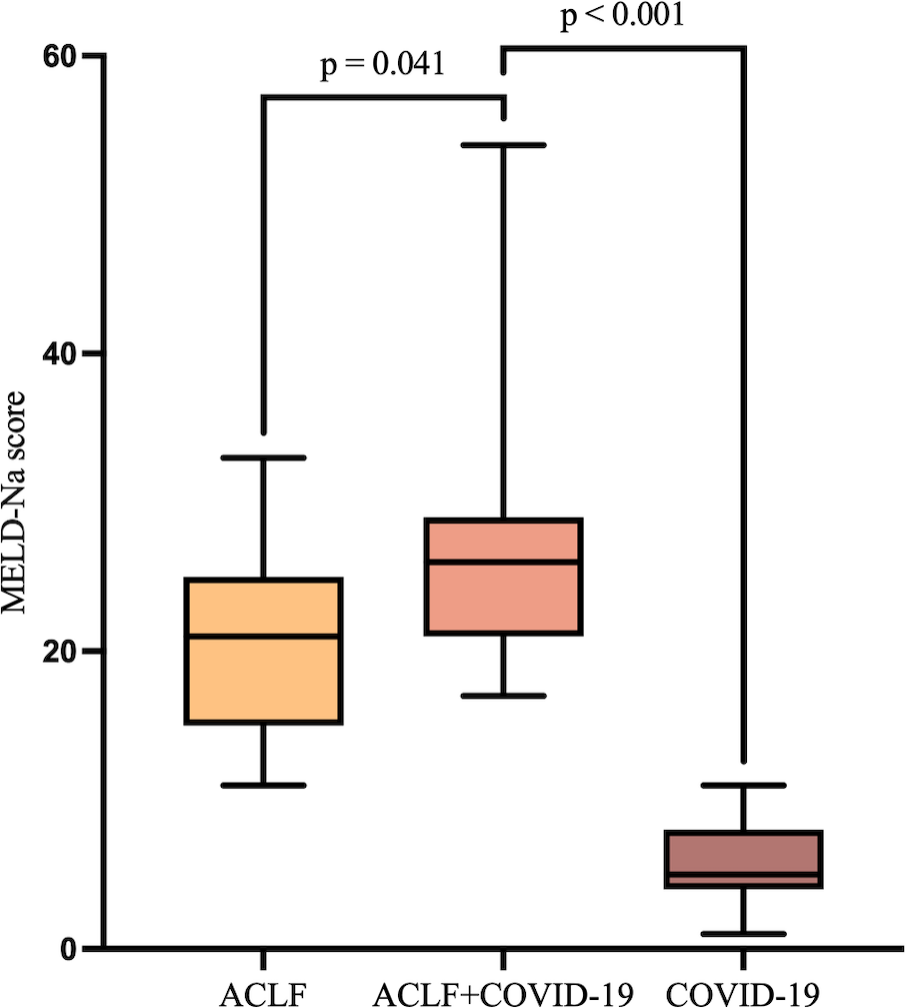
MELD-Na scores of patients in the ACLF, ACLF+COVID-19, and COVID-19 groups one week after admission.

**Figure 4:**
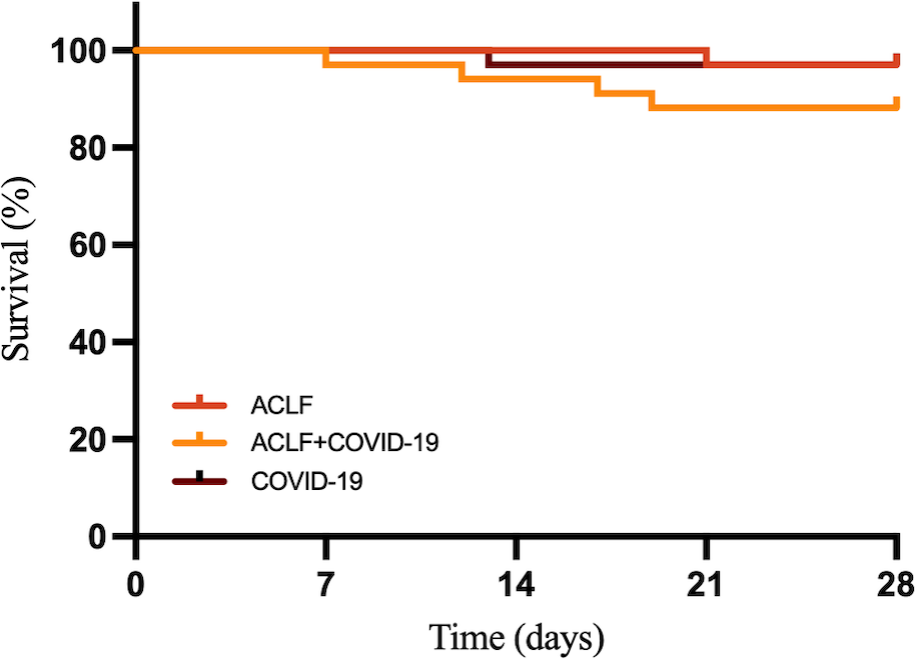
The 28-day survival curves of ACLF, ACLF+COVID-19, and COVID-19 groups.

The MELD-Na score showed no significant differences between the two ACLF groups at admission. However, it was significantly higher in the ACLF+COVID-19 group one week later (26 [21-29] *vs* 21 [15-25]; *p*=0.041), suggesting rapid disease progression within one week of admission (**Figure 3**). In addition, the MELD-Na score in the COVID-19 group was significantly lower than that in the ACLF+COVID-19 group one week after admission (26 [21-29] *vs* 5.87 ± 2.77; p <0.001). The 28-day mortality rate was slightly higher in the ACLF+COVID-19 group than in the ACLF group (4 [11.76%] vs 1 [2.9%]; p = 0.16) and the COVID-19 group (4 [11.76%] vs 1 [2.9%]; p = 0.16); however, these differences were not statistically significant (**Figure 4**).

## 4 Discussion

In the present multicenter study, ACLF patients with COVID-19 had an increased risk of thrombocytopenia, more pronounced inflammatory responses, and faster disease progression within 1 week of admission when compared with age-, gender-, etiology-, and MELD-Na score-matched ACLF patients without COVID-19. However, there was no significant increase in 28-day mortality.

Compared with the ACLF group, patients in the ACLF+COVID-19 group had more fever, cough, sputum production, fatigue, and hypoxemia. In addition, the proportion of patients with T2DM was higher in the ACLF+COVID-19 group, which was believed to be associated with the compromised immune system in diabetic patients (Pari et al., 2023). In particular, a larger proportion of ACLF+COVID-19 patients developed hepatic encephalopathy, suggesting that COVID-19 would predict a poor prognosis in patients with ACLF. The direct liver damage in COVID-19, coupled with the systemic inflammatory response elicited by the viral infection, may exacerbate the hepatic workload, leading to a decrement in hepatic functional reserve. This impairment hampers the liver’s capacity to adequately process toxins and metabolic byproducts, thereby potentially elevating the risk of developing hepatic encephalopathy.

Previous studies have shown that COVID-19 can induce liver injury (Cichoz-Lach and Michalak, 2021). Similarly, our present study showed that the ACLF+COVID-19 group had a more pronounced inflammatory response and a higher Meld-Na score at 1 week of admission compared with the ACLF group, which might be explained by the following three possible mechanisms. First, cytokine storm is one of the major causes of liver injury in COVID-19 patients (Effenberger et al., 2021). SARS-CoV-2 infection can trigger a cytokine storm by activating immune cells, thereby releasing large amounts of cytokines (eg, IL-6 and TNF-α) and leading to systemic inflammatory response syndrome (SIRS) and multi-organ dysfunction (MODS), which often affect the liver (Premkumar and Kedarisetty, 2021 ^;^ Naeem et al., 2023). Trebicka et al. suggested that IL-6 levels were closely related to the severity and prognosis of ACLF (Trebicka et al., 2019). In our present study, the ACLF+COVID-19 group had significantly higher IL-6 levels, and the IL-6 level was positively correlated with Meld-Na score, AARC score, and SOFA score. Additionally, the levels of IL-8 and IL-17 in the ACLF+COVID-19 group were significantly higher than those in the COVID-19 group. These findings further suggested that cytokine storm in COVID-19 aggravated liver damage. Second, SARS-CoV-2 enters cells by binding to ACE2 (Hoffmann et al., 2020 ^;^ Shang et al., 2020), thereafter causing cellular damage. Studies have shown that the liver also expresses ACE2 (Fiel et al., 2021 ^;^ Li et al., 2022). Thus, SARS-CoV-2 induces inflammation and cell apoptosis in the liver through ACE2 in liver tissue, which leads to deterioration of liver function. Third, hypoxemia can also exacerbate ischemia-hypoxia injury in the liver (Nardo et al., 2021 ^;^ Li et al., 2022). In our present study, hypoxemia was more pronounced in the ACLF+COVID-19 group than in the ACLF group. Therefore, the ACLF+COVID-19 group had higher Meld-Na scores at 1 week of admission, which might be that COVID-19 exacerbates liver damage through cytokine storm, direct effects of the virus, and hypoxemia.

In our study, COVID-19 did not significantly increase 28-day mortality rate in ACLF patients. Although patients in the ACLF+COVID-19 group had significantly higher MELD-Na scores at 1 week of admission, timely and effective treatments including antiviral therapy, artificial liver treatment, and supportive care reduced the cytokine storm and helped block disease progression. NBAL can reduce inflammatory factors, block cytokine storm, and improve survivals (Guo et al., 2020 ^;^ Dai et al., 2021). These early interventions might have effectively lowered the mortality rate in ACLF patients. Similar studies have demonstrated that hospitalized cirrhotic patients with COVID-19 did not face a higher mortality risk than those without COVID-19 (Bajaj et al., 2021), suggesting COVID-19 does not necessarily elevate the case-fatality rate in patients with hepatic insufficiency. Therefore, we speculate that the acute effects of COVID-19 infection may cause short-term liver function variability, with early intervention potentially lowering the mortality risk.

In our present study, patients in the ACLF+COVID-19 group had a significantly lower count of platelets compared to those in the ACLF group, and further stratification showed that patients with an AARC score grade II or III had significantly lower platelet count, suggesting that COVID-19 may worsen coagulation function in patients with ACLF, especially in critically ill ones. The liver is crucial for both the production and storage of platelets, and patients with liver damage often experience thrombocytopenia (Scharf, 2021). Reduced platelet count is not uncommon in patients with COVID-19. In a retrospective study of 1,099 COVID-19 patients, thrombocytopenia was noted in 36.2% of patients on admission (Terpos et al., 2020). A possible explanation is that the SARS-CoV-2 infection can directly invade hematopoietic stem cells, affecting their normal differentiation and proliferation; or, the virus-induced inflammatory response damages the bone marrow microenvironment and ultimately inhibits hematopoiesis (Zhang et al., 2020). COVID-19 has the potential to deteriorate liver function and thus influence platelet counts. As a result, patients with ACLF and COVID-19 have a significantly increased risk of developing thrombocytopenia. These patients are at greater risk of bleeding and are more likely to develop severe coagulopathy. Close monitoring of the changes in the platelet count and effective treatment of bleeding can improve the prognosis.

The present study revealed the clinical features of ACLF patients with COVID-19 but still had several limitations. First, the sample size was small. Nevertheless, as few studies have done in patients with COVID-19 and ACLF, our current study still provided useful clinical information for such patients. Second, this study failed to directly detect ACE2 expression in livers, mainly due to the poor coagulation in patients with liver failure. However, previous studies have well demonstrated that ACE2 is expressed in liver tissue. Third, while GM-CSF/G-CSF were found to be valuable for ACLF patients and COVID-19 patients, they had not been recommended by Chinese guidelines during the subject inclusion; as a result, we did not observe the efficacy of GM-CSF/G-CSF treatment. Our future studies will examine the role of GM-CSF/G-CSF in ACLF+COVID-19 patients as more clinical data emerge.

## 5 Conclusion

Patients with ACLF and COVID-19 have an increased risk of developing thrombocytopenia, more obvious inflammatory responses, and faster disease progression. Close monitoring and early adequate treatment are critical for these patients.

## Data Availability

The original contributions presented in the study are included in the article/supplementary material. Further inquiries can be directed to the corresponding authors.
