# Corrigendum: Clinical characteristics and the role of IL-6 in acute-on-chronic liver failure patients with or without COVID-19: a multicenter paired cohort study

**DOI:** 10.3389/fcimb.2025.1584105

**Published:** 2025-03-12

**Authors:** Ruoyu Yao, Guofen Xu, Xiujuan Fu, Wenrui Zhang, Han Wang, Yu Chen, Jia Yao

**Affiliations:** ^1^ Department of Gastroenterology, Third Hospital of Shanxi Medical University, Shanxi Bethune Hospital, Shanxi Academy of Medical Sciences, Tongji Shanxi Hospital, Taiyuan, China; ^2^ Fourth Department of Liver Disease (Difficult and Complicated Liver Diseases and Artificial Liver Center), Beijing You’an Hospital Affiliated to Capital Medical University, Beijing, China

**Keywords:** acute-on-chronic liver failure, COVID-19, SARS-CoV-2, mortality, prognosis, IL-6

In the published article, there was an error in **Figures 3** and **4** as published. The images for **Figures 3** and **4** were swapped in order. The corrected **Figures 3** and **4** and their captions appear below.

**Figure 3:**
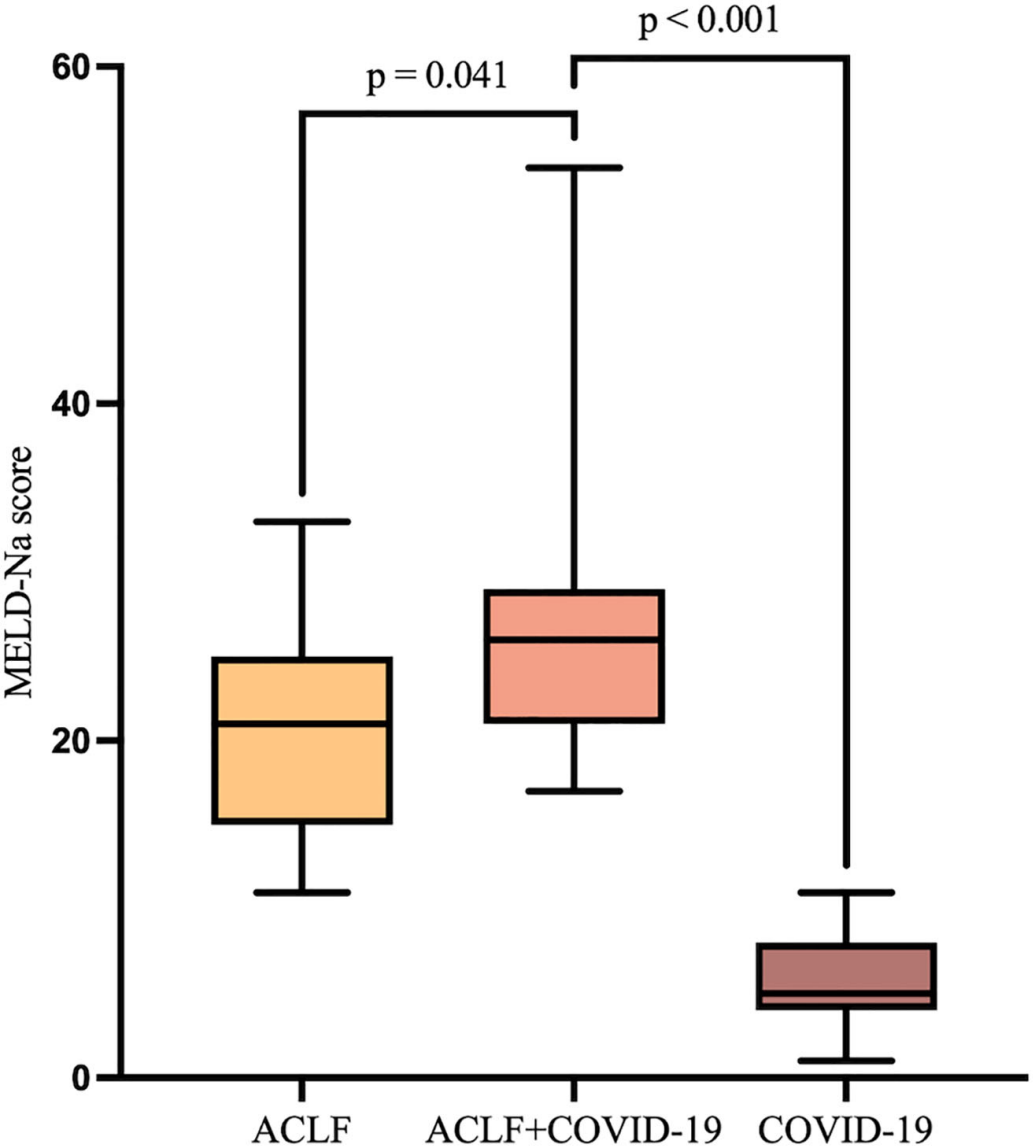
MELD-Na scores of patients in the ACLF, ACLF+COVID-19, and COVID-19 groups one week after admission.

**Figure 4:**
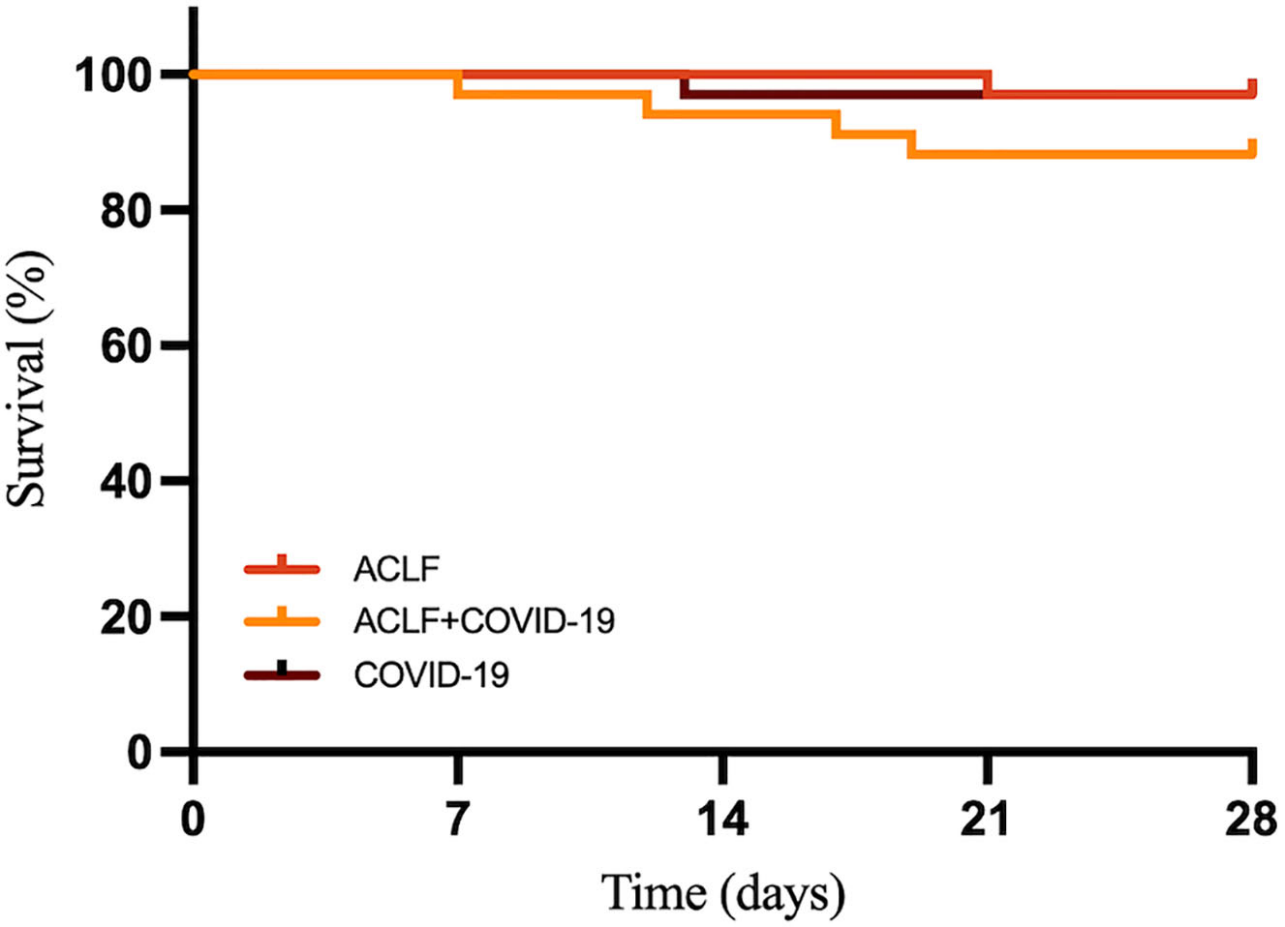
The 28-day survival curves of ACLF, ACLF+COVID-19, and COVID- 19 groups.

In the published article, there was an error in **Table 1** as published. The category headings for indicators were omitted in **Table 1**. The corrected **Table 1** and its caption appear below.

**Table 1:** Basic features of patients in the three groups at admission.

	ACLF group (n=34)	ACLF+COVID-19 group (n=34)	COVID-19 group (n=34)	^a^P-value	^b^P-value
Age, years	52 ± 9.67	52.82 ± 9.89	52.71 ± 10.01	0.73	0.769
Male, No. (%)	23 (67.65%)	23 (67.65%)	23 (67.65%)	1	1
Etiology					
HBV/Alcohol/Drug/AIH/Other, No.	11/16/2/3/2	11/16/1/3/3	–	1	–
Comorbidity					
T2DM, No. (%)	2 (5.88%)	8 (23.53%)	14 (41.18%)	0.04	0.12
Hypertension, No. (%)	8 (23.53%)	6 (17.65%)	12 (35.29%)	0.549	0.099
CVD, No. (%)	1 (2.94%)	2 (5.88%)	13 (38.24%)	1	0.001
Complication					
Ascites, No. (%)	29 (85.29%)	30 (88.24%)	0 (0%)	1	<0.001
HE, No. (%)	5 (14.71%)	14 (41.18%)	0 (0%)	0.015	<0.001
Blood Test Parameter					
ALT, IU/L	137.1 (42.2-377.7)	35.3 (21.93-348.5)	33.69 ± 24.67	0.191	0.165
AST, IU/L	328 (82.3-488.4)	113.2 (31.25-236)	26.7 (20.8-34.6)	0.237	0.001
TBIL, umol/L	390.84 ± 175.58	263.85 ± 188.11	15.03 ± 5.5	0.066	<0.001
DBIL, umol/L	288 (134.7-388.6)	173.9 (45.83-371.6)	3.2 (2.4-4.8)	0.101	<0.001
ALB, g/L	27.92 ± 3.49	30.07 ± 6.01	38.45 ± 5.03	0.242	<0.001
BUN, umol/L	4.2 (3.03-9.53)	8.65 (3.63-15.6)	5.63 ± 1.65	0.291	0.144
Cr, mmol/L	90 (75.4-125.1)	84.75 (70.5-141.3)	91.23 ± 25.84	0.917	0.548
K^+^, mmol/L	3.72 ± 0.8	3.83 ± 0.73	3.77 ± 0.26	0.682	0.761
Na^+^, mmol/L	133.79 ± 6.07	134.69 ± 5.26	136.34 ± 3.62	0.67	0.325
PT, s	22.39 ± 6.81	25.22 ± 5.34	11.8 (10.9-12.5)	0.215	<0.001
INR	1.91 (1.61-2.26)	2.36 (1.87-2.62)	1.09 (1.01-1.13)	0.174	<0.001
DD, ng/mL	1260 (953-3268)	1060.5 (309.25-3506.5)	169.13 ± 88.73	0.359	0.001
WBC, ×10^9^/L	7.12 ± 2.87	9.22 ± 6.04	6.21 ± 2.83	0.234	0.071
Hb, g/L	117.47 ± 30.09	98.47 ± 32.99	140.27 ± 14.69	0.111	<0.001
PLT, ×10^9^/L	90 (77-167)	55.5 (24-99)	180 (124-225)	0.016	<0.001
Neutrophil, ×10^9^/L	5.05 ± 2.54	7.54 ± 5.92	4.27 ± 2.25	0.145	0.029
Lymphocyte, ×10^9^/L	1.13 ± 0.59	0.86 ± 0.67	1.23 ± 0.82	0.254	0.11
Clinical Score					
MELD-Na score	26 ± 8	27 ± 13	8 ± 3	0.932	<0.001
AARC score	10 ± 2	10 ± 2	–	1	–
AARC score grade I	4 (11.76%)	3 (8.82%)	–	1	–
AARC score grade II	20 (58.82%)	20 (58.82%)	–	1	–
AARC score grade III	10 (29.41%)	11 (32.35%)	–	0.793	–
SOFA score	7 (6-9)	8 (7-9)	–	0.215	–

a P-value: Comparison between ACLF+COVID-19 group and ACLF group. ^b^P-value: Comparison between ACLF+COVID-19 group and COVID-19 group. HBV, hepatitis B virus; AIH, autoimmune hepatitis; T2DM, type 2 diabetes mellitus; CVD, cardiovascular disease; HE, hepatic encephalopathy; ALT, alanine aminotransferase; AST, aspartate aminotransferase; TBIL, total bilirubin; DBIL, direct bilirubin; ALB, albumin; BUN, blood urea nitrogen; Cr, creatinine; PT, prothrombin time; INR, international normalized ratio; DD, D-dimer; WBC, white blood cell; Hb, hemoglobin; PLT, platelets.

The authors apologize for this error and state that this does not change the scientific conclusions of the article in any way. The original article has been updated.

